# Effectiveness of Sublingual Immunotherapy in the Treatment of HDM-Induced Nasobronchial Allergies: A 3-Year Randomized Case-Control Study From Kashmir

**DOI:** 10.3389/fimmu.2021.723814

**Published:** 2021-10-13

**Authors:** Shahid M. Baba, Roohi Rasool, Ayaz Gull, Taha A. Qureshi, Afaq H. Beigh, Qurteeba Qadri, Zafar A. Shah

**Affiliations:** Department of Immunology and Molecular Medicine, Sher-i-Kashmir Institute of Medical Sciences, Srinagar, India

**Keywords:** asthma, allergic rhinitis, sublingual immunotherapy, skin prick test, Kashmir (India), House Dust Mites

## Abstract

Allergen immunotherapy (AIT) is the only disease-modifying treatment for allergic disorders that induces immunological tolerance through administration of specific allergens. Studies on AIT for subcutaneous route are in abundance; however, the efficacy of AIT in tablet form through sublingual route has not been well elucidated. The present prospective, parallel-group, controlled study sought to compare the efficacy of sublingual immunotherapy (SLIT) tablets with pharmacotherapy (PT) in 332 house dust mite (HDM)-specific allergic asthma and/or rhinitis patients over a period of 3 years. Patients were followed up for a 6-month run-in period and then randomly stratified as those who would receive SLIT, SLIT in addition to PT (SLIT+PT), and PT alone. AIT was administered in the form of sublingual tablets. Symptom and medication scores were measured every 3 months. *In vitro* evaluation of serum total and HDM specific immunoglobulin E (HDM sIgE) levels was carried out every 3 months, whereas *in vivo* skin prick test was performed annually for 3 years. Our study demonstrated sustained clinical improvement, reduction in inhaled corticosteroid (ICS) dose and duration as well as prevention from development of neosensitization to other aero allergens in HDM-allergic asthmatics and/or rhinitis patients treated with 3 years SLIT. Despite a remarkable clinical improvement with AIT, we observed that SLIT did not significantly change the skin reactivity to HDM at 3 years and there was no significant change in the ratio of serum total and HDM sIgE. Given the immune and disease modifying effects of AIT in allergic diseases, the present study supports the notion of its sublingual mode being an effective long-term immunomodulator in HDM-sensitized nasobronchial allergies.

## Introduction

Respiratory allergies, the prevalence of which has increased over the past 3 decades, affect a large part of the general population and have emerged as a global health problem (1, 2). Previous studies have shown that house dust mites (HDMs) *Dermatophagoides pteronyssinus* (D*p*) and *Dermatophagoides farinae* (D*f*) are the most common inhalant allergens worldwide that coexist in most geographical regions (3, 4). Approximately 85% of patients with respiratory allergies are typically HDM-allergic and if left untreated, can lead to an increased risk of asthma (3, 4). Environmental control and allergen avoidance measures have been found to be of limited applicability in reducing symptoms of allergic rhinitis and asthma (5, 6). While rescue treatment with antihistamines, nasal/inhaled corticosteroids (ICSs) and β_2_-agonists can control symptoms in majority of the patients, it does not modify the natural course of the disease and the underlying dysregulated immune response continues to be at work.

Allergen-specific immunotherapy (AIT) is the only etiology-based treatment modality capable of modifying disease manifestations due to its immunomodulatory effects and forms the cornerstone in the management of respiratory allergies (7–9). Unlike symptomatic drugs, AIT modulates the basic immunologic mechanism of the allergic disorders by providing specific and appropriate management that transforms the process of such diseases. Remarkably, AIT reduces the risk of new allergic sensitization and early treatment with specific immunotherapy can even prevent it from evolving into asthma (10, 11). Furthermore, the add-on advantage with AIT for HDM-induced respiratory allergies is long-term sustained clinical effect in posttreatment follow-up and halt in disease progression that is unlike with pharmacotherapy (PT) alone (12, 13). In the initial period of treatment, PT does assume its importance and needs to be given in unison with AIT, albeit to be subsequently replaced by AIT alone in a vast majority of the patients.

AIT administration through the subcutaneous route has been successfully carried out for decades. However, inconvenience of frequent hospital visits, discomfort of repeated injections and risk of life-threatening anaphylactic events have seen it being gradually replaced by new treatment modalities. Sublingual immunotherapy (SLIT) for HDM (SLIT-HDM) that involves applying allergen extract to the immunologically rich sublingual area has emerged as a safe daily and viable oral alternative to subcutaneous immunotherapy (SCIT). The World Health Organization-Allergic Rhinitis and its Impact on Asthma (ARIA) statement recommended the routine use of SLIT owing to its advantages of convenience of self-administration at home with a high compliance rate (14, 15). The precise immunological mechanisms underlying the beneficial clinical effects of SLIT are not yet clear. Induction of T regulatory lymphocytes that suppress the allergic response, production of blocking antibodies such as immunoglobulin G4 (IgG4) and IgA and the involvement of mucosal B cells are regarded as immunological markers of clinical tolerance induced by SLIT (16).

To the best of our knowledge, the current prospectively followed study is the first from North India to evaluate and compare the long-term efficacy of 3 years of SLIT on clinical and laboratory outcomes in HDM-monosensitized allergic rhinitis/allergic asthma patients and compare it with their peers on PT alone.

## Materials and Methods

### Study Subjects

This prospective, parallel-group and controlled study was conducted in the Department of Immunology and Molecular Medicine, Sher-i-Kashmir Institute of Medical Sciences (SKIMS). A total of 332 mild to moderate persistent allergic asthma and/or moderate to severe allergic rhinitis patients with skin prick test (SPT) confirmed diagnosis of HDM monosensitization by *in vivo*/*in vitro* specific immunoglobulin E (sIgE) testing and no previous history of immunotherapy, who attended the Allergy and Immunology Clinic of SKIMS were enrolled in the study. Patients with autoimmune disorders, other inflammatory disorders, malignancies and acute or chronic infectious diseases were excluded. This study was conducted only after taking informed consent from all participants or their legal guardians (for patients <18 years old) and approval from the local institutional ethics committee of Sher-i-Kashmir Institute of Medical Sciences, Kashmir, India (IEC-SKIMS) (Protocol No: RP:60/2018). All procedures performed in the present study were in compliance with the 1964 Declaration of Helsinki principles.

### Study Design

After a run-in period of 6 months mainly for evaluating their baseline symptom scores and to optimize medication scores, patients were randomized to receive SLIT, SLIT + PT, and PT only (control group) for at least 3 years and followed in the outpatient clinic. At baseline and defined treatment stages, all patients were evaluated by means of symptom and medication scores and skin prick test (SPT). Immunological markers like total IgE, HDM sIgE and serum vitamin D levels were evaluated at the same time points.

### Skin Prick Test

Skin prick testing was performed using common aeroallergens including dust mites, fungi, pollens, dust mix, animal epithelia and insect dander. Buffered saline and histamine phosphatase acted as negative and positive controls respectively. The test was done by introducing a drop of allergen extract into the skin *via* 1-mm allergy lancet on the back or volar side of the patient forearm. Cutaneous response was recorded 20 min after allergen challenge by measuring the diameter of the wheal reaction. A wheal size ≥3 mm was considered positive.

### Serum Total/House Dust Mite Specific Immunoglobulin E and Vitamin D Levels

Enzyme-linked immunosorbent assay (ELISA) technique was employed to quantify serum total IgE and vitamin D levels using specific kits (Boster Bio-Tech) as per the manufacturer’s instructions. Serum concentrations of total IgE and vitamin D were expressed in IU/ml and ng/ml respectively, and levels in the range of 0–180 for IgE and 30–100 for vitamin D were considered normal. ImmunoFluoriMetric assay (ImmunoCAP, Thermo Fisher Scientific, CA, USA) was used to determine HDM sIgE levels in serum. Concentrations of sIgE were expressed in kUA/l and levels over 0.35 kUA/l were considered positive.

### Pharmacologic Treatment and Allergen Avoidance

Patients were educated about specific allergen avoidance measures that include using allergen-proof mattress and pillow covers, washing bedding regularly with hot water, removing dust using damp duster, frequent vacuum cleaning with HEPA filter, removing soft toys from the bedrooms, keeping low humidity (below 60%) and avoiding upholstered furniture. Appropriate pharmacological treatment was initiated that included Intra nasal cortico steriod (INCS), oral antihistamines, oral leukotriene receptor blockers (LTRBs), inhaled corticosteroids (ICS) and long-acting beta-agonists (LABA). Patients were then reassessed monthly and the PT dose was accordingly modified. Oral drugs whenever indicated were required for only a few weeks. ICS-LABA and INS treatment was discontinued when the patient was asymptomatic for 3 months with minimal dose. Treatment groups were classified and compared according to the percentage of successful discontinuation of ICS at the end of 3 years of AIT. Successful discontinuation was defined as being asymptomatic after cessation of ICS treatment for at least 6 months.

### Allergen-Specific Immunotherapy

AIT was started in patients sensitive to HDM along with strong clinical correlation as per standard guidelines. The allergen SLIT formulation was provided by BAC pharmacy, Bangalore, India, and customized for each patient according to the individual sensitivity spectrum. The immunotherapy used in the present study was in tablet form bearing strength of 2,800 Biological Units (BU) of HDM (D*f*, D*p*, and Blomia in different ratios depending upon the score of each HDM component) and were given as once-a-day dosage. SLIT tablets were taken sublingually in the morning before breakfast, kept under the tongue for at least 5 min until it dissolved and mixed with saliva and was then swallowed subsequently. The duration of treatment was 3 years during which patients were followed up regularly.

### Symptom Score

Symptoms were assessed in every patient and scores were analyzed. Total duration of therapy was 36 months. All the patients were asked to complete a symptom questionnaire before immunotherapy and subsequently at every 1-year interval after receiving SLIT. The questionnaire for allergic rhinitis patients was based on Total Nasal Symptom Score (TNSS). The symptom score was recorded and averaged for 1 week using a 6-point scoring system (0, indicating no symptom; 1, very mild; 2, mild; 3, moderate; 4, severe; and 5, very severe). TNSS was defined as the sum of the scores for four nasal symptoms: rhinorrhea, sneezing, nasal obstruction and itchy nose (range, 0–20). For bronchial asthma patients, an Asthma Control Test (ACT) questionnaire was based on the symptom scores for breathlessness, wheezing and coughing, ranging from 0 to 25. Here, the 19 was baseline for well-controlled asthma, whereas any score below 19 was indicative of poorly controlled asthma. Patients were also asked to record their use of anti-allergic medications, such as oral antihistamine and intranasal/inhaled medication.

### Medication Score

Patients were made to record daily prophylactic and rescue medication taken by them on a diary card. A grading of 1 was given for taking β_2_-agonist on that day and 0 for not consuming any such drug. Similarly, for antihistamine intake such as cetirizine or loratadine for rhinitis, a score of 1 point was given. The daily dose of inhaled steroid was scored according to the micrograms of drug taken per day. For example, for inhaled budesonide, a score of 0 was given for not consuming the drug, 1 for taking 0–200 µg/day, 2 for taking 200–400 µg/day, 3 for taking 400–800 µg/day and 4 for taking 800–1,000 µg/day. Similarly, scores were given for intranasal steroids. As an example, for mometasone intake, the score was 0 for no drug intake, 1 for taking 50 µg/day, 2 for taking 100 µg/day and 3 for taking 200 µg/day. The combined medication score for allergic rhinitis and asthma was termed total medication score.

### Statistical Analysis

Statistical analysis was carried out by means of the SPSS Statistics for Windows, Version 25.0, released 2017 (IBM Corp., New York, USA). Values were presented as means (SD), unless otherwise specified. Comparisons between groups were tested for significance using chi-square and paired t-tests. p-value <0.05 was defined to be statistically significant.

## Results

The present study enrolled 332 cases comprised of 168 allergic rhinitis and 164 allergic asthma patients who were stratified into three treatment groups. Among them, 164 patients received SLIT only, 88 received SLIT in addition to pharmacological treatment (SLIT+PT), whereas 80 patients received PT only. SLIT+PT and PT groups were further categorized into three subgroups on the basis of type of allergic disorder and pharmacological regimen used. Out of 168 patients in the SLIT+PT and PT groups combined, 81 patients had allergic rhinitis and were managed with daily dosage of intranasal mometasone 50 μg and for a few patients, oral LTRB (montelukast 10 mg) and antihistamine (H1 blocker, e.g., cetirizine 10 mg) were given. Furthermore, 80 patients with allergic asthma were treated with daily dosage of inhalational budesonide (200/400 μg) and formoterol (6 μg). The groups also contained seven patients with allergic rhinitis plus allergic asthma and were managed with combination therapy used for the treatment of the other two subgroups (**Supplementary Figure S1**). The mean age of patients in SLIT group was 31 ± 15.28 years, 29 ± 12.70 years for SLIT+PT group, and for PT group, the mean age was 33 ± 15.88 years. Across the three treatment groups female patients were more prevalent compared to males. Demographic features indicated that the proportion of rural and urban patients were similar among the three groups (**Table 1**). The duration of AIT was 36 months. There were 14 dropouts from the SLIT group (six at the end of the first year, three after the second year and four at the end of the third year) because of noncompliance with treatment, failure to follow-up and financial reasons. Similarly, eight patients (three each at first and second year, two after the third year) dropped out from the SLIT+PT, whereas five patients each withdrew at the end of the second and third year in the PT controls.

**Table 1:** Demographic features of allergic patients.

Parameters	SLIT (n = 164)	SLIT+PT (n = 88)	PT (n = 80)	p-value
**Age**	31 ± 15.28	29 ± 12.70	33 ± 15.88	0.35
**Gender**				
Male	69 (42.1%)	36 (40.9%)	33 (41.2%)	0.99
Female	95 (57.9%)	52 (59.1%)	47 (58.8%)	
**Dwelling**				
Rural	76 (46.3%)	50 (56.8%)	37 (46.3%)	0.18
Urban	88 (53.7%)	38 (43.2%)	43 (53.7%)	
**Diagnosis**				
Allergic rhinitis	94 (57.3%)	28 (31.8%)	46 (57.5%)	—
Allergic asthma	70 (42.7%)	60 (68.2%)	34 (42.5%)	—
**Duration of AIT**				
**in months**	36	36	—	—

PT, pharmacotherapy; SLIT, sublingual immunotherapy.

### Effect of Sublingual Immunotherapy on Symptom Score

**Figure 1:**
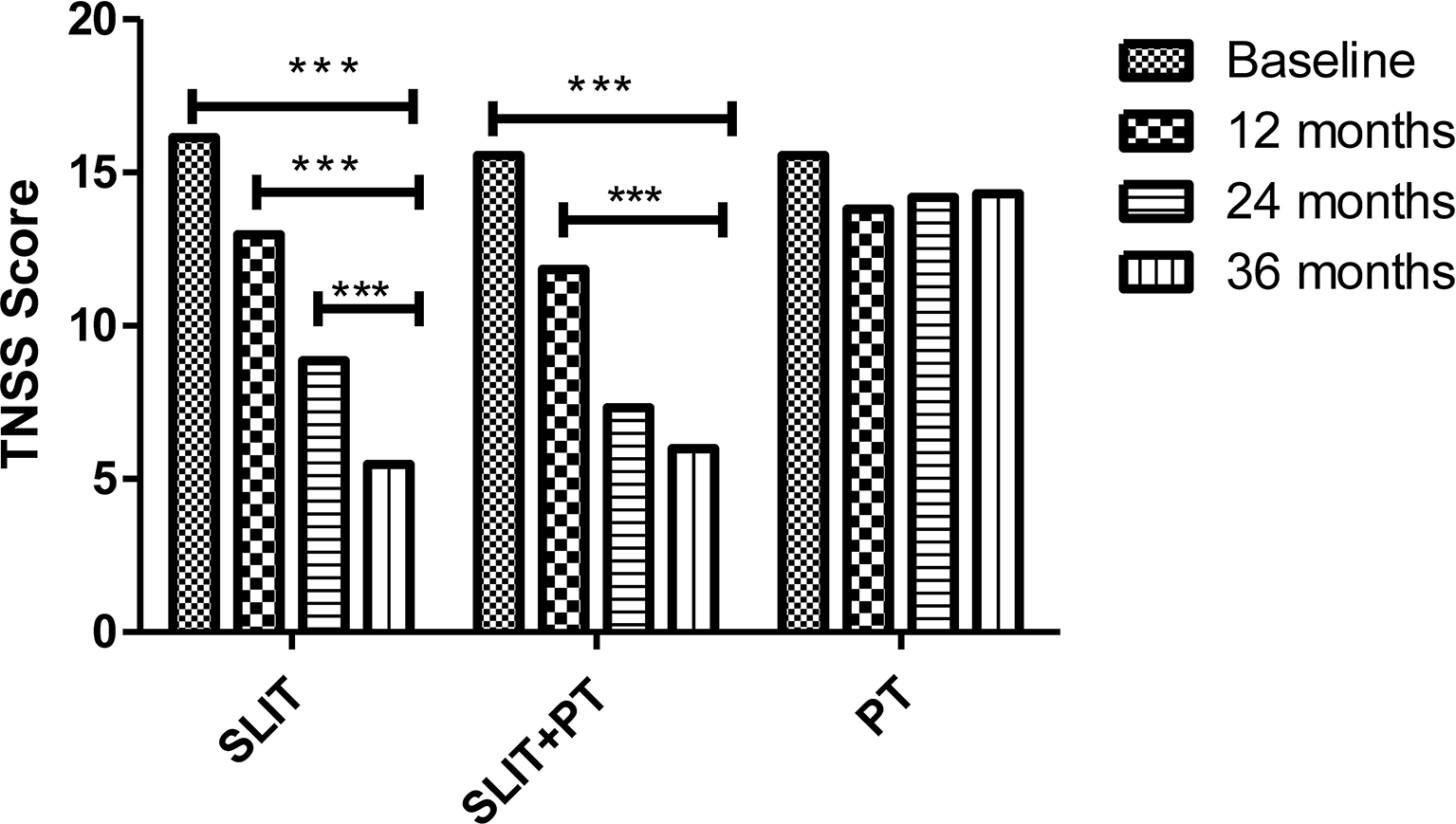
Total Nasal Symptoms Score (TNSS) of 3 treatment groups at different stages. *** denotes level of statistical significance.

**Figure 2:**
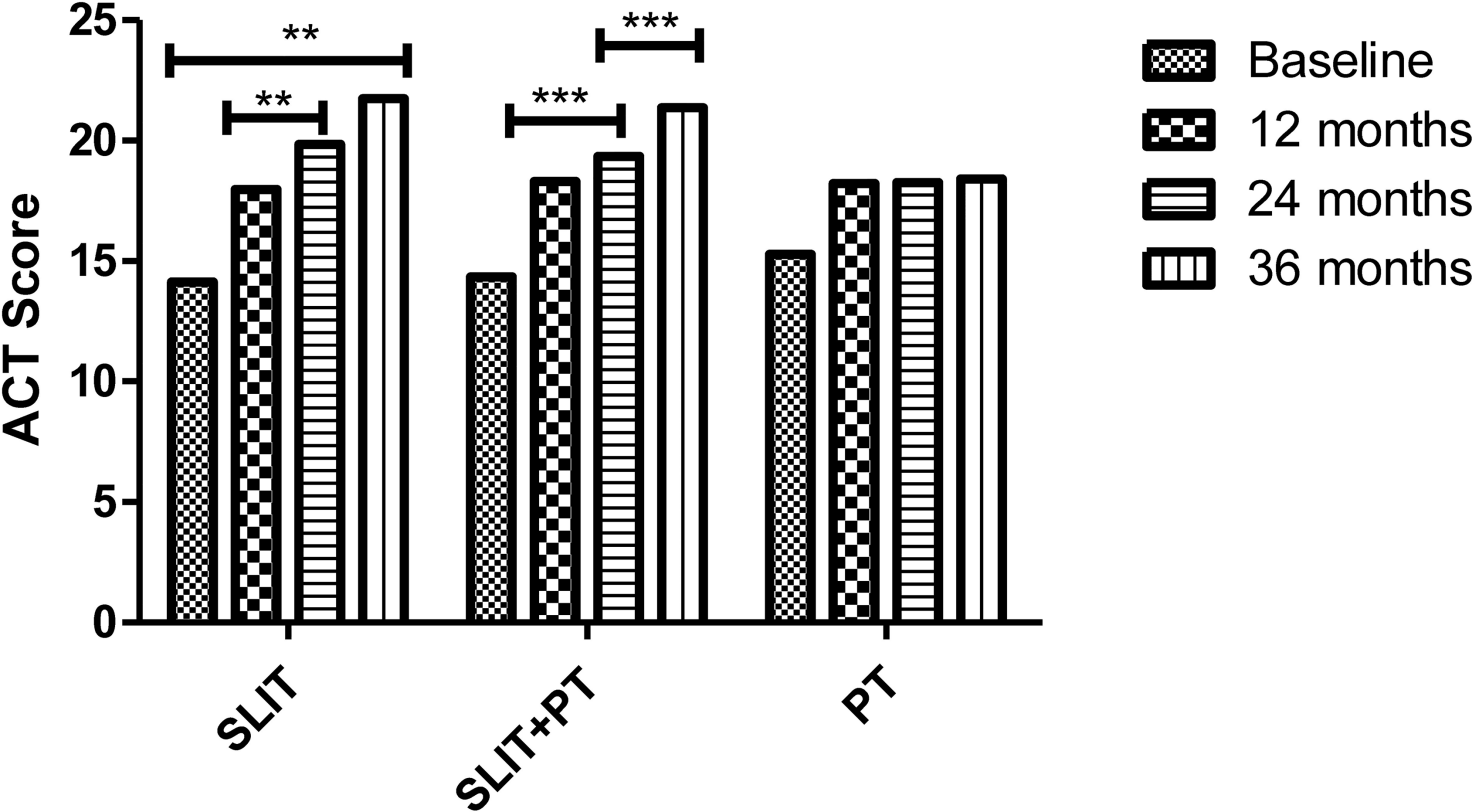
Asthma Control Test (ACT) of three treatment groups at different stages. ** denotes level of statistical significance which was p = 0.006. *** denotes level of statistical significance.

The primary endpoints of our study were assessing improvement in clinical symptom scores TNSS and ACT and the secondary endpoints were measuring parameters like skin reactivity to allergens, serum total IgE and HDM sIgE across the three treatment groups after 3 years of follow-up. SLIT significantly reduced TNSS score at 1 year (12.97 ± 4.18), 24 months (8.85 ± 1.35), and 36 months (5.47 ± 1.65) compared to (16.14 ± 7.23) at baseline (p = 0.001). Similarly, for SLIT+PT group, a significant gradual decrease in symptom score with increasing time duration was observed (11.84 ± 3.65 after 12 months, 7.32 ± 2.33 after 24 months, 5.99 ± 2.23 after 36 months) as compared to baseline (15.65 ± 5.34) (p = 0.002). On the contrary, no significant changes in symptom scores were observed in PT-only group (p = 0.56) (**Figure 1**). We observed a significant improvement in ACT of the patients with bronchial asthma at 12, 24, and 36 months (17.97 ± 5.65, 19.85 ± 7.42, 21.74 ± 7.35, respectively) after taking SLIT compared to baseline (14.14 ± 6.12) (p = 0.006). In case of SLIT+PT group, the ACT of patients also improved significantly after 3 years of treatment (21.36 ± 6.73) compared with baseline (14.35 ± 3.65) (p = 0.007), but no significant changes were observed in the control group (p > 0.05) (**Figure 2**).

### Effect of Sublingual Immunotherapy on Medication Score

**Figure 3:**
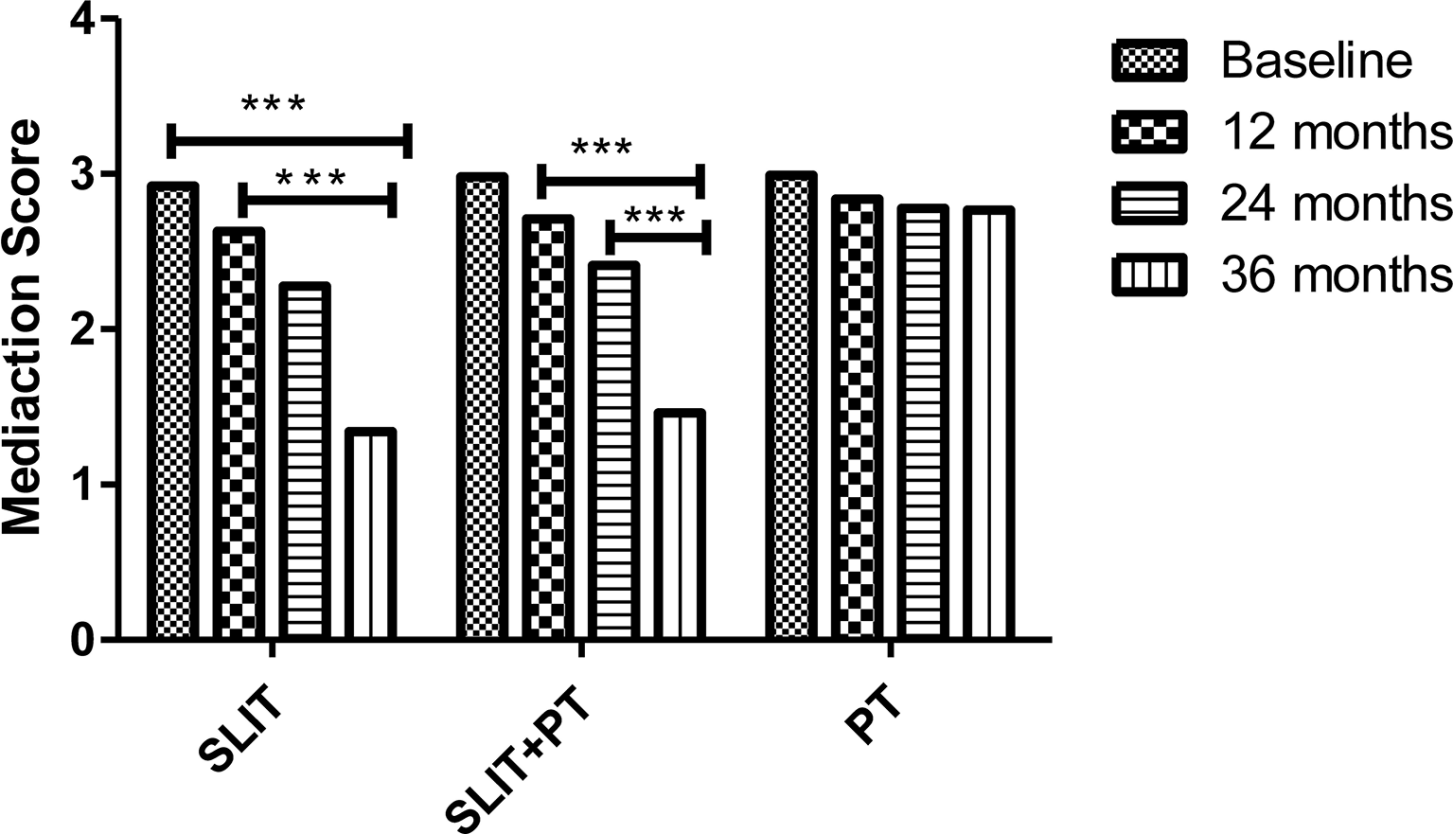
Medication scores of three treatment groups at different stages. *** denotes level of statistical significance.

The effect of allergen immunotherapy on the two stratified treatment groups was analyzed by assessing change in their respective medication score during the course of our study and compare with the group that received PT only. In SLIT group, there was a significant reduction in the medication score at the end of 12, 24, and 36 months of treatment (2.63 ± 0.19, 2.28 ± 0.44, 1.34 ± 0.23, respectively) as compared to baseline (2.92 ± 0.22) (p = 0.02). Similarly, a significant reduction in the annual medication score was observed for SLIT+PT group after 3 years of treatment compared to baseline (1.46 ± 0.44 *vs.* 2.98 ± 0.14) (p = 0.03). Meanwhile, no significant decrease in annual medication score was detected at the end of the study period in the PT group (p > 0.05) (**Figure 3**).

### Effect of Sublingual Immunotherapy on Corticosteroid Dosage

We observed a significant gradual reduction in the annual dosage of ICS treatment in SLIT+PT group after 3 years of follow-up (p = 0.001). Whereas in the PT group, although the mean daily dose of ICS decreased at the end of the second and third year, the difference was not significant (p = 0.54) **(** **Table 2** **)**.

**Table 2:** Decrease in ICS intake after 3 years of AIT.

ICS intake	Baseline	12 months	24 months	36 months	p-value
**SLIT+PT**	340 ± 90	310 ± 76	280 ± 64	210 ± 58	**0.001**
**PT**	330 ± 87	376 ± 93	340 ± 67	390 ± 101	0.54

AIT, allergen-specific immunotherapy; ICS, inhaled corticosteroid; PT, pharmacotherapy; SLIT, sublingual immunotherapy. Bold denotes statistically significant values.

### Serological Markers

The analysis of allergy markers revealed no significant changes in serum total IgE levels posttreatment in any of the three groups studied. Furthermore, neither SLIT nor the SLIT+PT or PT group showed any significant changes in HDM sIgE. All the three groups enrolled in the study were vitamin D-deficient at baseline. Both inter- and intra-group comparisons revealed no significant difference in serum vitamin D levels at defined treatment stages (**Table 3**).

**Table 3:** Effect of 3 years of AIT on serum markers.

Serum Markers	Baseline	12 months	24 months	36 months	p-value
**Total IgE**					
SLIT	453 ± 80	526 ± 95	498 ± 110	482 ± 98	0.52
SLIT+PT	477 ± 103	510 ± 153	493 ± 123	461 ± 109	0.87
PT	493 ± 93	489 ± 140	486 ± 97	488 ± 132	0.54
**HDM sIgE**					
SLIT	40 ± 18	47 ± 19	43 ± 24	38 ± 22	0.47
SLIT+PT	42 ± 21	45 ± 18	39 ± 20	37 ± 16	0.33
PT	39.9 ± 15	38 ± 15	37.6 ± 18	37.1 ± 22	0.49
**Vitamin D**					
SLIT	15.22 ± 3.34	18.34 ± 10.5	26.32 ± 12.12	29.44 ± 12.21	0.56
SLIT+PT	15.32 ± 7.22	15.43 ± 5.22	17.87 ± 10.32	25.54 ± 6.33	0.72
PT	14.58 ± 5.43	18.65 ± 8.31	22.32 ± 5.77	26.43 ± 5.23	0.62

AIT, allergen-specific immunotherapy; HDM, house dust mite; PT, pharmacotherapy; sIgE, specific immunoglobulin E; SLIT, sublingual immunotherapy.

### Skin Prick Test Reactivity

**Figure 4:**
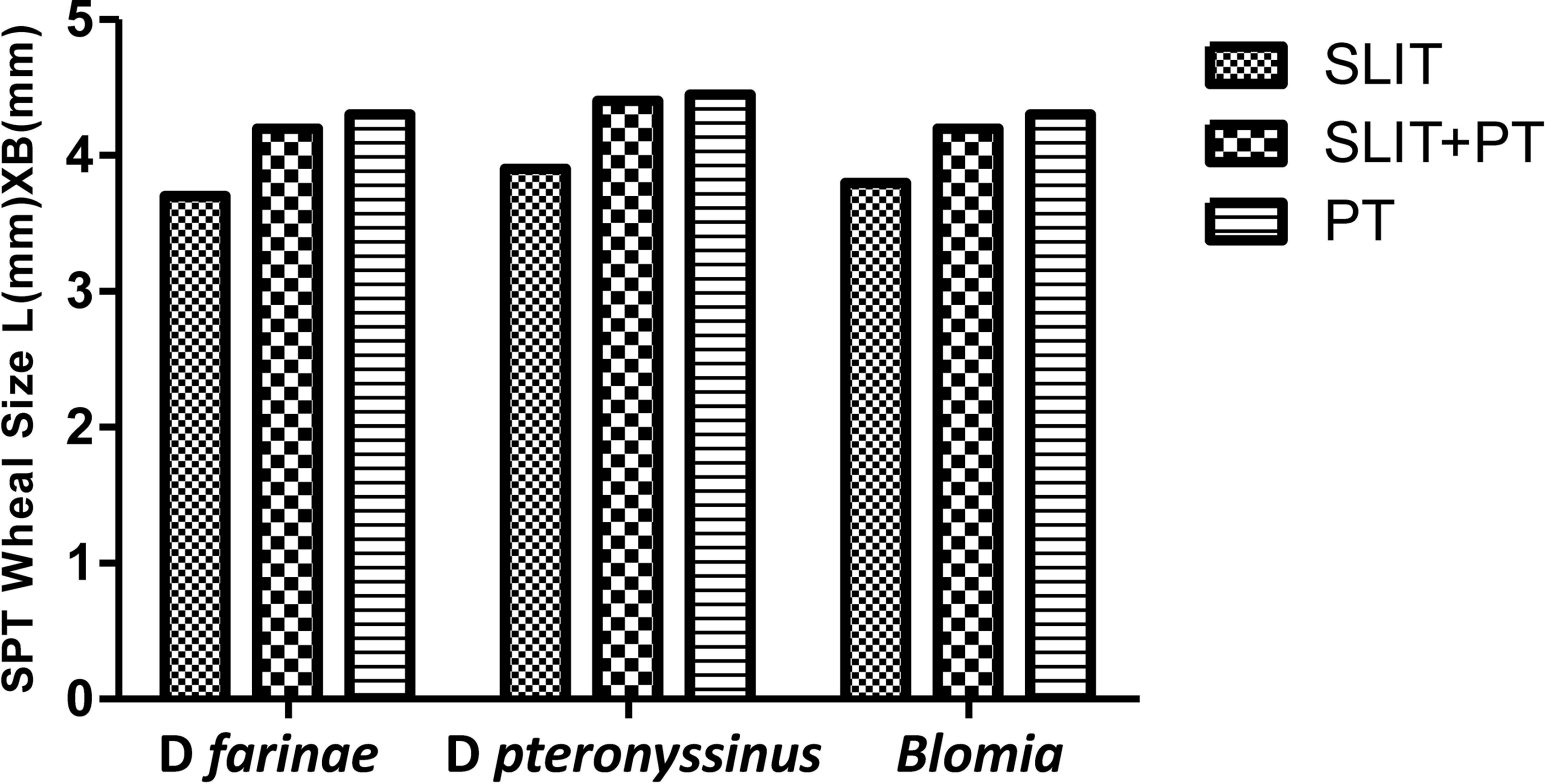
Wheal diameter values of skin prick test (SPT) to house dust mite (HDM) across three treatment groups at different stages.

We observed no difference in skin reactivity to D*f*, D*p*, and Blomia in either group after 3 years of follow-up (p > 0.05). Also, inter-group comparisons revealed no new sensitizations at the end of 3 years of treatment (**Figure 4**).

### Side Effects

None of the patients in the present study developed any acute exacerbations requiring emergency hospitalization during the course of SLIT.

## Discussion

Treatment of respiratory allergies such as allergic rhinitis and asthma by PT is an effective and safe treatment. However, many of these standard drugs have not been tested specifically in the context of HDM allergy, resulting in only poor to moderate symptom control (17). AIT represents the only current treatment with a potential to modify the course of allergic respiratory diseases by inducing immunological tolerance to the causal allergen. SLIT has been extensively investigated in respiratory allergic diseases during the last two decades and its efficacy has been confirmed by many controlled studies (14, 15, 18–21). SLIT is rapidly gaining physician acceptance and patient adherence as well as compliance because of not only its efficacy but also its safety profile and the convenience of administration. Furthermore, the amount of allergen administered *via* sublingual tablets is 100-fold more than that delivered by subcutaneous injections, thereby establishing tolerance faster. The build-up phase required in SCIT is usually shortened or eliminated in SLIT (22). The studies have also reported that the effects of SLIT last for at least 7–12 years after treatment discontinuation (8).

In the current prospective, randomized, long-term follow-up controlled study, we evaluated the steroid-sparing effect of 3 years of SLIT in patients with HDM-related allergic rhinitis and asthma. For this study, patients after proper diagnosis were treated with regular ICS regimen and followed up for a run-in period of 6 months. Patients were then categorized as those who continued pharmacologic treatment only, those who received SLIT only and those who received SLIT in addition to pharmacologic treatment for 3 years.

The efficacy and long-term effect of HDM-SLIT for treatment of patients with allergic rhinitis and asthma have been demonstrated in both children and adults in many prospective studies (23–27). In addition, large-scale controlled trials of SLIT with other allergens have confirmed the long-term clinical efficacy and disease-modifying effect after 3 years of treatment with SLIT (28).

Our study demonstrates that SLIT for HDM allergy showed better clinical improvement and significantly reduced both rhinitis/asthma symptoms and medication usage compared to cases treated with PT alone. At the end of 3 years, we observed that SLIT alone or in unison with PT reduced rhinitis symptoms by 71.65% and 70.05% respectively, and asthma symptoms by 13.74% and 13.04% respectively.

Also, the medication scores of our study group decreased significantly in SLIT group and SLIT+PT group. The percent decrease was found to be higher in SLIT group (55.34%) followed by SLIT+PT group (51.34%) and least percentage decrease was noted in PT-only group (15.67%). We also observed a significant decrease in annual duration and mean daily dose of ICS in the SLIT and SLIT+PT groups, but not in controls. Our results are consistent with the findings of many recently published studies in different populations across the world (25–27). Karakoc-Aydiner et al. (26) reported improved clinical outcomes in children with HDM-driven allergic rhinitis and/or asthma after 3 years of treatment with SLIT compared to PT only. The study also reported a better safety profile for SLIT than SCIT (26). Furthermore, Ayfer et al. (25), through a double-blind and double-dummy design study, demonstrated enhanced clinical efficacy of SLIT on reducing the symptoms of allergic rhinitis and asthma after the 1-year treatment compared to the baseline. In addition, Ozdemir et al. (27) reported a significant reduction in the dosage as well as duration of ICSs and its successful discontinuation for at least 6 months along with improvement in lung functions in children with HDM-allergic asthma after 3 years of SLIT in conjunction with PT compared with cases who were treated with PT alone.

Our results showed no significant difference in skin reactivity to HDM as well as no neosensitization across the three study groups after 3 years of treatment, which is consistent with the findings of Ozdemir et al. (27), who also reported no significant changes in inter- or intra-group skin reactivity to HDM during follow-up of 3 years. In contrast, studies by Ayfer et al. (25) and Karakoc-Aydiner et al. (26) demonstrated a significant decrease in wheal diameter of D*p* and D*f* extracts in the immunotherapy groups after 3 years of treatment compared with baseline and control group. Meanwhile, there is not much data available regarding development of new sensitizations for SLIT.

IgE levels often increase in patients with allergic rhinitis and/or allergic asthma who are naturally exposed to specific allergens. Also, after initiation of allergen immunotherapy, the concentrations of allergen-specific IgE rise and then gradually decrease over months or years to the pretreatment levels or lower (29). The present study observed no significant changes in total serum IgE, HDM sIgE, and serum vitamin D levels in HDM-sensitized allergic rhinitis and asthmatic patients across the three treatment groups at the end of the study. A study similar to ours by Karakoc-Aydiner et al. (26) also reported no significant changes in serum total IgE or HDM sIgE in patients after 3 years of treatment. On the contrary, a number of studies have reported a significant decrease in both serum IgE and HDM sIgE levels at the end of 3 years of treatment with SLIT (25, 27). In sync with many previous studies, we recorded no serious adverse reactions during the course of our study that might have resulted in treatment discontinuation.

Only a limited number of studies across the globe have explored clinical efficacy and immunological outcomes of HDM-SLIT. To the best of our knowledge, this is the first randomized, prospective, 3-parallel grouped study from India regarding HDM-related allergic rhinitis/asthma patients treated with SLIT and followed prospectively for 3 years. The design, selection of HDM-monosensitized patients, initial 6-month run-in period before the administration of SLIT and long-term follow-up providing details of changes in clinical and immunological aspects induced by immunotherapy increased the strength of the present study. A double-blind, placebo-controlled study design would have further enhanced the power of the study, but for ethical and practical reasons, it was not feasible.

## Conclusion

In conclusion, we, through this study, demonstrated improved clinical outcomes and reduction/discontinuation of both the duration and dose of PT in HDM-monosensitized patients with asthma and/or rhinitis treated with SLIT for 3 years compared to PT-only group. Moreover, we also attempted to show that SLIT can prevent the development of neosensitization. Further large clinical studies should be conducted to more precisely determine the sustained long-term effects of SLIT in chronic persistent allergic rhinitis/asthma as we enter the age of precision medicine.

## Data Availability Statement

The datasets presented in this article will be made available to anyone on a proper request to the corresponding author. Requests to access the datasets should be directed to shahidbaba3512@gmail.com.

## Ethics Statement

The studies involving human participants were reviewed and approved by Institutional Ethics Committee of Sher-i-Kashmir Institute of Medical Sciences. Written informed consent to participate in this study was provided by the patient or by the participants’ legal guardian.

## Author Contributions

SB conceptualized and designed the study, did lab work, and drafted the manuscript. RR supervised the study, recruited patients, provided logistical support, and submitted the article. AG performed lab work. TQ recruited patients and administered AIT. AB and QQ compiled data and did statistical work. ZS did proofreading and manuscript correction. All authors contributed to the article and approved the submitted version.

## Conflict of Interest

The authors declare that the research was conducted in the absence of any commercial or financial relationships that could be construed as a potential conflict of interest.

## Publisher’s Note

All claims expressed in this article are solely those of the authors and do not necessarily represent those of their affiliated organizations, or those of the publisher, the editors and the reviewers. Any product that may be evaluated in this article, or claim that may be made by its manufacturer, is not guaranteed or endorsed by the publisher.
